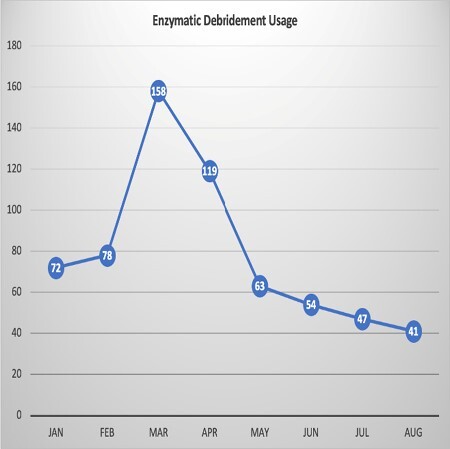# 715 Initial Use of PLA Skin Substitute at a Large Public Safety-Net Hospital

**DOI:** 10.1093/jbcr/irae036.259

**Published:** 2024-04-17

**Authors:** Jessica Delamater, Larisa Shagabayeva, Michael Cobler-Lichter, Brianna L Collie, Nicole B Lyons, Shevonne S Satahoo, Joyce I Kaufman, Louis R Pizano, Carl I Schulman

**Affiliations:** University of Miami/Jackson Memorial Hospital, Miami, FL; University of Miami, Fort Lauderdale, FL; DeWitt Daughtry Family Department of Surgery, University of Miami, Miami, FL; University of Miami/Jackson Memorial Hospital, Miami, FL; University of Miami, Fort Lauderdale, FL; DeWitt Daughtry Family Department of Surgery, University of Miami, Miami, FL; University of Miami/Jackson Memorial Hospital, Miami, FL; University of Miami, Fort Lauderdale, FL; DeWitt Daughtry Family Department of Surgery, University of Miami, Miami, FL; University of Miami/Jackson Memorial Hospital, Miami, FL; University of Miami, Fort Lauderdale, FL; DeWitt Daughtry Family Department of Surgery, University of Miami, Miami, FL; University of Miami/Jackson Memorial Hospital, Miami, FL; University of Miami, Fort Lauderdale, FL; DeWitt Daughtry Family Department of Surgery, University of Miami, Miami, FL; University of Miami/Jackson Memorial Hospital, Miami, FL; University of Miami, Fort Lauderdale, FL; DeWitt Daughtry Family Department of Surgery, University of Miami, Miami, FL; University of Miami/Jackson Memorial Hospital, Miami, FL; University of Miami, Fort Lauderdale, FL; DeWitt Daughtry Family Department of Surgery, University of Miami, Miami, FL; University of Miami/Jackson Memorial Hospital, Miami, FL; University of Miami, Fort Lauderdale, FL; DeWitt Daughtry Family Department of Surgery, University of Miami, Miami, FL; University of Miami/Jackson Memorial Hospital, Miami, FL; University of Miami, Fort Lauderdale, FL; DeWitt Daughtry Family Department of Surgery, University of Miami, Miami, FL

## Abstract

**Introduction:**

The loss of available xenograft as a skin substitute posed significant problems for our burn patients with partial thickness burns and the inability to transition to outpatient wound care or with intractable pain. This led to increasing length of stay and increased utilization of resources. The public hospital system was also challenged with additional unique patient problems that were left without a good solution. We hypothesized that the introduction and use of a PLA skin substitute had decreased costs and utilization of resources after its introduction into a large public safety-net hospital.

**Methods:**

We began using a PLA skin substitute in April 2022. The hospital length of stay for patients admitted to the burn floor was compared for 3 months before and after the introduction of the PLA skin substitute. To account for the transition and ramp up of use, January through March were compared to June through August, thereby excluding April and May as a transition period. In addition, the utilization of enzymatic debridement and its cost were also compared for 3 months before and after the PLA skin substitute was introduced.

**Results:**

The number of tubes of enzymatic debridement went down from 308 to 142 with an associated cost savings of $38,000. Additionally, the length of stay decreased from an average of 13.5 days to 8.9 days. Given the cost of a single hospital day this extrapolates to a yearly savings of over $200,000. It was also noted that certain patient populations that traditionally had a difficult time transitioning to outpatient care, such as homeless patients and international patients, were more quickly discharged after PLA skin substitute placement.

**Conclusions:**

This study demonstrated a decrease in both resource utilization and costs very quickly after the introduction of a PLA skin substitute in a large public safety net hospital. Future studies need to look at longer times before and after the introduction of the skin substitute to validate that these reductions are sustained.

**Applicability of Research to Practice:**

These findings suggest both patients and health care systems may benefit from more widespread use of a PLA skin substitute in the treatment of burn patients.